# Scaling Surgical Resources: A Capacity Analysis of C-arm Machines in Haiti Following the 2021 Earthquake

**DOI:** 10.1007/s00268-023-06958-x

**Published:** 2023-03-08

**Authors:** Helena Franco, Samuel Simister, Abdoulie Njai, Pierre Marie Woolley, Michelle Nyah Joseph

**Affiliations:** 1 Department of Global Health and Social Medicine, Harvard Medical School, Boston, MA USA; 2 Bond University, Gold Coast, QLD Australia; 3 Program in Global Surgery and Social Change, Harvard Medical School, Boston, MA USA; 4 School of Medicine, University of Utah, Salt Lake City, UT USA; 5 School of Business, University of Utah, Salt Lake City, UT USA; 6 Harvard T.H Chan School of Public Health, Boston, MA USA; 7 School of Medicine, University of Missouri-Columbia, Columbia, MO USA; 8 La Paix State University Hospital UEH, Port-au-Prince, Haiti; 9 School of Medicine, Université Notre Dame d’Haïti, Port-au-Prince, Haiti; 10 Clinical Trials Unit, University of Warwick, Warwickshire, UK

## Abstract

**Background:**

In 2021, a 7.2 magnitude earthquake struck Haiti resulting in a surge of orthopaedic trauma requiring immediate surgical treatment. Safe and efficient operative management of orthopaedic trauma injuries requires intraoperative fluoroscopy through C-arm machines. The Haitian Health Network (HHN) received a philanthropic donation of three C-arm machines and considered an analytical tool may guide efficacious placement of those machines. The study objective was to develop and apply a clinical needs and hospital readiness measuring tool relevant to C-arm machines, which may guide decision-makers, such as HHN, in response to an emergency situation with a surge in need for orthopaedic treatment.

**Methods:**

An online survey to assess surgical volume and capacity was created and then completed by a senior surgeon or hospital administrator based at hospitals within the HHN. Multiple-choice and free-text answer data were collected and classified into five categories: staff, space, stuff, systems, and surgical capacity. Each hospital received a final score out of 100, calculated by equal weighting of each category.

**Results:**

Ten out of twelve hospitals completed the survey. The average weighted score for the staff category was 10.2 (SD 5.12), the space category was 13.1 (SD 4.09), the stuff category was 15.6 (SD 2.56), the systems category was 12.25 (SD 6.50), and the surgical capacity category was 9.5 (SD 6.47). The average final hospital scores ranged from 29.5 to 83.0.

**Conclusion:**

This analysis tool provided data as to the clinical demand and capabilities of hospitals within the HHN to receive a C-arm machine and reaffirmed the critical need for more C-arms in Haiti. This methodology may be utilised by other health systems to provide data to distribute orthopaedic trauma equipment, which would benefit communities during periods of surge capacity, such as natural disasters.

**Supplementary Information:**

The online version contains supplementary material available at 10.1007/s00268-023-06958-x.

## Introduction

The Republic of Haiti, the third largest country in the Caribbean, experiences ongoing political instability, poor health infrastructure, and recurrent natural disasters such as 7.0 earthquake in 2010 and Hurricane Matthew in 2016 [1]. On 14 August 2021, a 7.2 magnitude earthquake struck Les Cayes, 150 km west of Port-au-Prince [2], resulting in an estimated death of more than 2,200 people [3]. The impact of the earthquake restricted access to rural areas and caused widespread destruction of health facilities and utility infrastructure further burdening the healthcare system [1]. Compounded with political deterioration and natural disasters such as Tropical Storm Grace, the earthquake exacerbated pre-existing need for improved surgical services and significantly increased the need for critical orthopaedic trauma interventions.

While the full extent of injuries sustained in the earthquake remains unknown, initial reports suggested at least 12,000 people were injured in the immediate earthquake [1], including a significant number of limb injuries requiring orthopaedic surgical management [2]. There are three options for receiving surgical treatment in Haiti: public hospitals, private hospitals in metropolitan areas that require a financial contribution, and charitable hospitals [4]. Proper surgical management of orthopaedic injuries often necessitates intraoperative imaging via a C-arm machine (C-arm). A C-arm is a portable medical imaging device that utilises X-ray technology to allow intraoperative radiography and fluoroscopy during orthopaedic trauma and elective surgery. There are three components: an X-ray generator, an imaging system, and a workstation unit. As intraoperative devices, these machines allow surgeons to monitor the progress of orthopaedic operations, including fracture reduction and fixation, to improve anatomical alignment, reduce operative time, reduce intraoperative complications such as blood loss, and reduce post-operative infection. Furthermore, intraoperating imaging can be stored to mitigate the requirement for post-operative radiographs [5–7]. The World Health Organization Guidelines for Essential Trauma Care (WHO GETC) recognises that C-arm machines are an “integral part of orthopaedic armamentarium” and despite being classified as desired due to the associated cost suggests that high-volume hospitals should consider C-arm machines as essential [8].

Within Haiti, the 2021 earthquake provided an example of how natural disasters result in an acute surge of surgical trauma and strain on healthcare capacity. Surgical injuries after the earthquake were mainly orthopaedic, including open fractures, closed fractures, and crush injuries [9, 10]. In the aftermath of the earthquake, the Haiti Health Network (HHN) identified eleven existing C-arms. Only three C-arm machines were fully operational, of which two were located in the Northern region, approximately 170 km from the earthquake epicentre and did not receive any patients following the earthquake. The remaining eight non-functional C-arm machines required biomedical engineering inspection, repairs, or servicing, which would necessitate many technical hours with no guarantee of safe operability. In the context of increased demand in orthopaedic trauma capacity, the lack of functional C-arms may delay treatment, prolong surgical time, increase the risk of revision surgery, and limit surgical treatment options [11].

Three GE OEC 9600 C-arm machine systems were donated to Haiti in the aftermath of the earthquake. As there is no existing framework for assessing clinical need and hospital readiness for C-arm allocation, the Haitian Health Network identified a need for a tool to guide the efficacious placement of these machines. The aim of this study was to perform a baseline capacity analysis of the twelve HHN hospitals to determine the clinical need, orthopaedic trauma operative caseload requiring C-arms, and hospital readiness to operate and maintain C-arm machines, and thus to create a template for future similar situations.

## Material and methods

### Study design

Approval was obtained by the authors’ institution’ Human Research Ethics Committee (IRB21-1467) and by the local Ethics Board as a retrospective capacity and impact quantitative data collection.

### Participants

Hospitals and health services within the HHN with a functional operating room facility that provides surgical trauma care for orthopaedic trauma by a trained orthopaedic surgeon were invited to participate. Consent was voluntarily obtained from the institution at the commencement of the survey. The survey was distributed to the twelve hospitals that met the inclusion criteria identified through the Dalton Foundation who work with the HHN.

### Setting and survey tool

The online survey (Suppl. 1) was designed using Qualtrics XM in English and French and was open for completion between 1 January 2022 and 31 March 2022. At each hospital, a senior surgeon or hospital administrator completed the survey. The survey was divided into five categories of free-text and multiple-choice questions: staff, space, stuff, systems, and surgical capacity, as detailed in Suppl. 2. Within this study, “staff” was defined as staff relevant to operation and maintenance of C-arm machines, “space” was defined as hospital facilities used for C-arms, “stuff” was defined as radiological and surgical equipment and hospital infrastructure related to C-arms, “systems” was defined as processes for C-arm induction, operation, and maintenance, and “surgical capacity” was defined as the hospitals’ orthopaedic surgical caseload. Each of these categories contained variables relevant to the operation and maintenance of C-arm machines for orthopaedic trauma surgery. These variables were constructed from previous capacity surgical tools, the WHO GETC [8], trauma assessment tools in adjacent fields [12–15], and using the authors’ clinical experience in Haiti and orthopaedic trauma.

Within the five categories, variables were designated by the authors to one of two subcategories: (1) “essential”, for the functioning of a C-arm machine or (2) “desired”, for the functioning of a C-arm machine. The full list of variables according to each category and subcategory is contained in Suppl. 2. The staff category had 45 variables, including 39 essential and six desired. The space category had two essential variables and no desired variables. The stuff category had 24 variables, being 20 essential and four desired. The systems had nine variables, which were all essential. Finally, the surgical capacity category had twelve variables, which were all essential.

### Statistical analysis

The statistical analysis was performed using Microsoft Excel (Seattle, Washington, USA). Data were recorded and tabulated into the research database for each hospital. The survey tool only collected quantitative data of a continuous and count nature. The responses were de-identified before the research team completed the analysis. To obtain final scores for each hospital, several steps were necessary:

1. Tabulating the variables to produce a raw score for each of the five categories.

Responses for the essential subcategory were tabulated and multiplied by two. Responses for the desired subcategories were tabulated and multiplied by one. The totals of each were combined to produce a raw score for each of the five categories, being “raw staff”, “raw space”, “raw stuff”, “raw systems”, and “raw surgical capacity” scores. This was repeated for each of the hospitals.

(2) Weighting of raw category scores to produce a weighted score for each of the five categories.

The raw staff scores for all hospitals were listed, and the highest raw staff score was identified. For each hospital, the raw staff score was divided by the highest raw staff score. This value was weighted to represent 20% of the final hospital score by being multiplied by 20 (“weighted staff score”). This was repeated to calculate weighted space, weighted stuff, weighted systems, and weighted surgical capacity scores for each hospital.

(3) Adding weighted category scores to produce a final score for each hospital.

The weighted staff, weighted space, weighted stuff, weighted systems, and weighted surgical capacity scores were added to produce a final score for each hospital. These final scores, ranked from highest to lowest, created a list of hospitals, which was provided to the Dalton Foundation to make recommendations on where the three additional donated C-arm machines were placed.

## Results

Ten hospitals (83.3%) completed the online survey between 1 January 2022 and 31 March 2022. All online surveys were fully completed. The survey tally and raw scores for the essential and desired subcategories are summarised in Table 1. Of these ten hospitals, three hospitals reported having a functional C-arm machine (30%) and two hospitals (20%) reported having a non-functional C-arm machine. The weighted category scores and final score for each hospital are shown in Table 2.

**Table 1:** Raw scores for each category (staff, space, stuff, systems, and surgical capacity)

Hospital	One	Two	Three	Four	Five	Six	Seven	Eight	Nine	Ten
*Staff*										
Tally-essential	24	50	45	20	23	34	17	14	20	12
Tally-desired	4	14	6	10	5	14	4	3	0	4
Raw-essentials	48	100	90	40	46	68	34	28	40	24
Raw-desired	4	14	6	10	5	14	4	3	0	4
Raw score (total)	52	114	96	50	51	82	38	31	40	28
*Space*										
Tally-essential	5	6	7	5	3	6	4	3	3	4
Tally-desired	–	–	–	–	–	–	–	–	–	–
Raw-essentials	10	12	14	10	6	12	8	6	6	8
Raw-desired	–	–	–	–	–	–	–	–	–	–
Raw score (total)	10	12	14	10	6	12	8	6	6	8
*Stuff*										
Tally-essential	66	55	57	50	57	64	48	47	56	41
Tally-desired	8	2	8	6	2	18	2	1	7	6
Raw-essentials	132	110	114	100	114	128	96	94	112	82
Raw-desired	8	2	8	6	2	18	2	1	7	6
Raw score (total)	140	112	122	106	116	146	98	95	119	88
*Systems*										
Tally-essential	8	4	8	1	5	7	4	1	7	4
Tally-desired	–	–	–	–	–	–	–	–	–	–
Raw-essentials	16	8	16	2	10	14	8	2	14	8
Raw-desired	–	–	–	–	–	–	–	–	–	–
Raw score (total)	16	8	16	2	10	14	8	2	14	8
*Surgery*										
Tally-essential	8	7	19	20	11	14	6	0	6	4
Tally-desired	–	–	–	–	–	–	–	–	–	–
Raw-essentials	16	14	19	40	22	28	12	0	12	8
Raw-desired	–	–	–	–	–	–	–	–	–	–
Raw score (total)	16	14	38	40	22	28	12	0	12	8

**Table 2:** Weighted category scores and final score for each hospital

Hospital	Staff	Space	Stuff	Systems	Surgical capacity	Final score
One	9.1	14.3	19.2	20.0	8.0	70.6
Two	20.0	17.1	15.3	10.0	7.0	69.5
Three	16.8	20.0	16.7	20.0	19.0	92.6
Four	8.8	14.3	14.5	2.5	20.0	60.1
Five	8.9	8.6	15.9	12.5	11.0	56.9
Six	14.4	17.1	20.0	17.5	14.0	83.0
Seven	6.7	11.4	13.4	10.0	6.0	47.5
Eight	5.4	8.6	13.0	2.5	0.0	29.5
Nine	7.0	8.6	16.3	17.5	6.0	55.4
Ten	4.9	11.4	12.1	10.0	4.0	42.4

### Weighted category score results

Within the staff category, the average weighted score was 10.2 (range 4.9–20.0; SD, 5.12). Within the space category, the average weighted score was 13.1 (range 8.6–20.0; SD, 4.09). Within the stuff category, the average weighted score was 15.6 (range 12.1–20.0; SD, 2.56). Within the systems category, the average weighted score was 12.25 (range 2.5–20.0; SD 6.50). Within the surgical capacity category, the average weighted score was 9.5 (range 0–20.0; SD 6.47).

### Final hospital score results

The final hospital scores ranged from 29.5 (hospital eight) to 83.0 (hospital three) with a standard deviation of 18.89. Of the ten included hospitals, hospitals three, six, and four reported having a functioning C-arm machine.

## Discussion

This study discusses a novel baseline capacity analysis framework for hospitals to rapidly assess the clinical need and hospital readiness to operate and maintain C-arm machines, which was created after the 2021 earthquake in Haiti. This framework was then adopted to calculate a score for ten hospitals to determine the allocation of three C-arms donated to the HHN by a philanthropic group through an arrangement external to this study. To the authors’ knowledge, this is the first time in the literature that any framework has been proposed to assess the capacity of C-arms in a low- and middle-income setting or following significant trauma.

While there have not been previous assessment tools described in the literature on the assessment of surgical capacity following natural disasters, studies have described surgical and anaesthetic capacity across several countries using the World Health Organization Tool for Situational Analysis to Assess Emergency and Essential Surgical Care (WHO Tool) [16] or the WHO GETC [8]. Specific to the orthopaedic and trauma field, Agarwal-Harding et al. [17] created a capacity survey tool for initial management, definitive treatment and aftercare for traumatic diaphyseal femoral fractures in Malawi, based on the WHO GETC and clinical experience. While no studies have directly reported a tool to assess clinical need and hospital readiness for C-arm machines, Chokotho et al. [18] conducted a baseline analysis of trauma and musculoskeletal care across East, Central, and Southern Africa, reporting that only 25% of referring hospitals had access to a functioning C-arm.

As the first study to describe a survey tool to determine the readiness and need of hospitals for C-arm machines, this methodology expanded on the WHO GETC to recognise other factors contributing to hospitals’ ability to operate and maintain a C-arm. Factors such as radiological technicians, protective lead apparel to prevent radiation exposure, and maintenance capabilities were included. The survey tool was created in close collaboration with Haiti’s local orthopaedic teams. Positive feedback was received by the HHN and Dalton Foundation for the final scores reflecting the perceived readiness of the hospitals to receive a C-arm machine, which may be considered an indirect external validation for the framework described.

The framework provided data relevant to the allocation of three C-arm machines donated to the HHN by a philanthropic group through an arrangement external to this study. Whilst the final decision-making process of the HHN is outside the scope of this paper, several approaches may be adopted to utilise the data. The first possible approach is for the three hospitals with the highest scores, hospitals three, six, and one, to be selected. This would provide the C-arm machines to the three hospitals best placed to deliver orthopaedic care. The second possible approach is for the three hospitals with the highest scores without a currently functional C-arm machine, hospitals one, two, and five, to be selected. This would provide the C-arm machines to the three hospitals with a robust hospital system to deliver orthopaedic care but would also prioritise the hospitals currently without functional C-arm machines. The final possible approach is to select the three hospitals with the lowest scores without a currently functional C-arm machine, which are hospitals eight, ten, and seven. This approach would prioritise hospitals that are currently the most poorly resourced, with the expectation that a C-arm machine would significantly benefit those hospitals, and provide the hospitals with a means to increase their resources. In the case of concerns about the hospital’s capacity to successfully operate a C-arm machine, only essential variables could be considered.

The 2010 earthquake in Haiti highlighted the requirement for the improvement of surgical systems, particularly orthopaedic trauma, to prevent complications such as revision surgeries [19]. Haiti is prone to natural disasters; however, there is also a baseline level of orthopaedic trauma secondary to road traffic injuries and increasing civil unrest, resulting in high incidence of orthopaedic trauma [1]. The clinical need and readiness survey described through this methodology allow health networks, hospitals, and philanthropic groups to advocate for resources and system support to manage such strain. Additionally, these data may be used to measure change across different hospitals. More broadly, trauma is the leading cause of death and disability within lower- and middle-income countries [20], with considerable recent efforts to improve trauma care. While this survey was developed for Haiti, it may provide data to enhance orthopaedic trauma management more broadly and during times of increased surgical strain. Healthcare systems and networks may experience such increases due to increased patient volume, increased acuity, particular care demands, or reduced resources [21], or during natural disasters and conflict [22]. Therefore, tools, such as the methodology provided, are an opportunity to provide a snapshot of hospitals to determine the clinical need and readiness for C-arm machines to improve the quality of orthopaedic care.

### Limitations

This study is limited by the small number of facilities surveyed, being ten out of the twelve eligible facilities in Haiti. Only one representative at each facility, either the senior surgeon or hospital administrator, completed the corresponding survey. Thus, our information is limited by potential self-selecting responder bias as there may have been an element of social desirability with respondents either over-reporting or under-reporting the need and readiness of the hospital facility. Given the urgency for distribution of the equipment following the earthquake, the variables were drawn from the literature and the authors’ clinical knowledge. To further improve the tool, a panel of experts could have been engaged in survey development to include more medical practitioners and allied health professionals with experience managing orthopaedic trauma in Haiti or other low-resource settings.

## Conclusion

The surge in orthopaedic trauma resulting from natural disasters presents challenges in healthcare capacity among low-resource settings. In the present study, a baseline capacity methodology was created to provide data concerning the hospitals’ needs and capabilities for this equipment. This methodology may provide a framework for future philanthropic groups to evaluate surgical capacity with the end goal of providing aid to overwhelmed surgical systems.

## Supplementary Information

Below is the link to the electronic supplementary material.

Supplementary file1 (DOCX 30 KB)
