# Supplementary material for: Scaling Surgical Resources: A Capacity Analysis of C-arm Machines in Haiti Following the 2021 Earthquake
Source: World J Surg. 2023 Mar 8;47(6):1419–25. doi: 10.1007/s00268-023-06958-x (PMC10156824; doi:10.1007/s00268-023-06958-x)
Supplement: Supplementary file 1 — Supplementary file1 (DOCX 30 KB) [file 268_2023_6958_MOESM1_ESM.docx]

**Title: Scaling Surgical Resources: A capacity analysis of C-arm Machines in Haiti following the 2021 Earthquake**

**Journal name: World Journal of Surgery**

**Authors:**

Helena Franco, MD, MSurg ^1,2,3^

Sam Simister ^3,4,5^

Abdoulie Njai, MPH ^3,6,7^

Pierre Marie Woolley, MD ^8,9^

Michelle Nyah Joseph, MBBS, BSc(Hons), MSc, PhD, FRCS ^1,3,10^

Affiliations:

^1^ Harvard Medical School, Department of Global Health and Social Medicine, Boston, Massachusetts, United States of America

^2^ Bond University, Gold Coast, Queensland, Australia

^3^ Harvard Medical School, Program in Global Surgery and Social Change, Boston, Massachusetts, United States of America

^4^ University of Utah, School of Medicine, Salt Lake City, Utah, United States of America

^5^ University of Utah, School of Business, United States of America

^6^ Harvard T.H Chan School of Public Health, Boston, Massachusetts, United States of America

^7^ University of Missouri-Columbia, School of Medicine, Columbia, Missouri, United States of America

^8^ La Paix State University Hospital UEH, Port-au-Prince, Haiti

^9^ Université Notre Dame d’Haïti, School of Medicine, Port-au-Prince, Haiti

^10^ Clinical Trials Unit, University of Warwick, Warwickshire, United Kingdom

Full addresses:

Helena Franco, Harvard Medical School, Department of Global Health and Social Medicine, Boston, Massachusetts, United States of America

Sam Simister, University of Utah, School of Medicine, Salt Lake City, Utah, United States of America

Abdoulie Naji, University of Missouri, Columbia School of Medicine, Columbia, Missouri, United States of America

Pierre Woolley, Université Notre Dame d’Haïti, School of Medicine, Port-au-Prince, Haiti

Michelle Joseph, Harvard Medical School, Program in Global Surgery and Social Change, United States of America

**Details for corresponding author:**

Helena Franco

Email: Helena_Franco@hms.harvard.edu

**Supplementary material 1: Survey to determine the capacity of hospitals for a C-arm machine**

Q1 – What is the name of the facility?

Q2 – What is your position/role at the hospital?

Q3 – How many General surgeons work at your facility?

1. Full time (5 days/week)
2. Part time (2-4 days/week)
3. Part time (1 day/week)
4. Haitian visiting surgeon
5. Foreign visiting surgeon

Q4 – How many Obstetric and Gynaecology surgeons work at your facility?

1. Full time (5 days/week)
2. Part time (2-4 days/week)
3. Part time (1 day/week)
4. Haitian visiting surgeon
5. Foreign visiting surgeon

Q5 – How many Orthopaedic surgeons work at your facility?

1. Full time (5 days/week)
2. Part time (2-4 days/week)
3. Part time (1 day/week)
4. Haitian visiting surgeon
5. Foreign visiting surgeon

Q6 – How many Ear, Nose and Throat surgeons work at your facility?

1. Full time (5 days/week)
2. Part time (2-4 days/week)
3. Part time (1 day/week)
4. Haitian visiting surgeon
5. Foreign visiting surgeon

Q7 – How many Paediatric surgeons work at your facility?

1. Full time (5 days/week)
2. Part time (2-4 days/week)
3. Part time (1 day/week)
4. Haitian visiting surgeon
5. Foreign visiting surgeon

Q8 – How many Orthopaedic Residents are at your facility?

1. Haitian Full time (5 days/week)
2. Haitian Part time (2-4 days/week)
3. Haitian Part time (1 day/week)
4. Foreign visiting resident

Q9 – How many other surgeons are at your facility?

1. Full time (5 days/week)
2. Part time (2-4 days/week)
3. Part time (1 day/week)
4. Haitian visiting surgeon
5. Foreign visiting surgeon

Q10 – How many Biomedical Technicians work at your facility?

1. Full time (5 days/week)
2. Part time (2-4 days/week)
3. Part time (1 day/week)
4. Haitian visiting biomedical technician
5. Foreign visiting biomedical technician

Q11 – Please select the option that best describes your source of electricity in the operating room

1. Electricity from a city/village source
   1. Never: less than 10% of time
   2. Rarely: 25-49% of time
   3. Seldom: 25-49% of time
   4. Sometimes: 50-74% of time
   5. Most: 75-100% of time
2. Electricity from a generator
   1. Never: less than 10% of time
   2. Rarely: 25-49% of time
   3. Seldom: 25-49% of time
   4. Sometimes: 50-74% of time
   5. Most: 75-100% of time
3. Electricity from a solar source
   1. Never: less than 10% of time
   2. Rarely: 25-49% of time
   3. Seldom: 25-49% of time
   4. Sometimes: 50-74% of time
   5. Most: 75-100% of time

Q12 – Please select the option that best describes your internet connection in the operating room

1. Never: less than 10% of time
2. Rarely: 25-49% of time
3. Seldom: 25-49% of time
4. Sometimes: 50-74% of time
5. Most: 75-100% of time

Q13 – Please select the option that best describes your generator

1. Fuel and fuel storage to keep generator running
   1. Never: less than 10% of time
   2. Rarely: 25-49% of time
   3. Seldom: 25-49% of time
   4. Sometimes: 50-74% of time
   5. Most: 75-100% of time
2. Person to repair generator
   1. Never: less than 10% of time
   2. Rarely: 25-49% of time
   3. Seldom: 25-49% of time
   4. Sometimes: 50-74% of time
   5. Most: 75-100% of time

Q14 – Please select the option that best describes the equipment available at your facility

1. X-ray with film processing
   1. Never: less than 10% of time
   2. Rarely: 25-49% of time
   3. Seldom: 25-49% of time
   4. Sometimes: 50-74% of time
   5. Most: 75-100% of time
2. X-ray with computed radiography processing
   1. Never: less than 10% of time
   2. Rarely: 25-49% of time
   3. Seldom: 25-49% of time
   4. Sometimes: 50-74% of time
   5. Most: 75-100% of time
3. X-ray with digital processing
   1. Never: less than 10% of time
   2. Rarely: 25-49% of time
   3. Seldom: 25-49% of time
   4. Sometimes: 50-74% of time
   5. Most: 75-100% of time
4. Orthopaedic operating table
   1. Never: less than 10% of time
   2. Rarely: 25-49% of time
   3. Seldom: 25-49% of time
   4. Sometimes: 50-74% of time
   5. Most: 75-100% of time
5. Orthopaedic drill and accessories
   1. Never: less than 10% of time
   2. Rarely: 25-49% of time
   3. Seldom: 25-49% of time
   4. Sometimes: 50-74% of time
   5. Most: 75-100% of time
6. Sterilizer for orthopaedic instruments
   1. Never: less than 10% of time
   2. Rarely: 25-49% of time
   3. Seldom: 25-49% of time
   4. Sometimes: 50-74% of time
   5. Most: 75-100% of time
7. Orthopaedic kits and hardware
   1. Never: less than 10% of time
   2. Rarely: 25-49% of time
   3. Seldom: 25-49% of time
   4. Sometimes: 50-74% of time
   5. Most: 75-100% of time
8. C-arm machines
   1. Never: less than 10% of time
   2. Rarely: 25-49% of time
   3. Seldom: 25-49% of time
   4. Sometimes: 50-74% of time
   5. Most: 75-100% of time

Q15 – How many orthopaedic surgeries do you perform at your facility every month?

Q16 – How many of these orthopaedic surgeries require imaging?

Q17 – What often do you perform the following surgeries at your hospital every month?

1. Open reduction and internal fixation
   1. 1-5 procedures per month
   2. 6-10 procedures per month
   3. 11-15 procedures per month
   4. 16-20 procedures per month
   5. 21-25 procedures per month
   6. Over 25 procedures per month
2. Closed reduction and internal fixation
   1. 1-5 procedures per month
   2. 6-10 procedures per month
   3. 11-15 procedures per month
   4. 16-20 procedures per month
   5. 21-25 procedures per month
   6. Over 25 procedures per month
3. External fixation of the upper and/or lower limb
   1. 1-5 procedures per month
   2. 6-10 procedures per month
   3. 11-15 procedures per month
   4. 16-20 procedures per month
   5. 21-25 procedures per month
   6. Over 25 procedures per month
4. K-wiring
   1. 1-5 procedures per month
   2. 6-10 procedures per month
   3. 11-15 procedures per month
   4. 16-20 procedures per month
   5. 21-25 procedures per month
   6. Over 25 procedures per month
5. Plating
   1. 1-5 procedures per month
   2. 6-10 procedures per month
   3. 11-15 procedures per month
   4. 16-20 procedures per month
   5. 21-25 procedures per month
   6. Over 25 procedures per month
6. Closed reduction and application of plaster
   1. 1-5 procedures per month
   2. 6-10 procedures per month
   3. 11-15 procedures per month
   4. 16-20 procedures per month
   5. 21-25 procedures per month
   6. Over 25 procedures per month
7. Closed reduction and internal wire
   1. 1-5 procedures per month
   2. 6-10 procedures per month
   3. 11-15 procedures per month
   4. 16-20 procedures per month
   5. 21-25 procedures per month
   6. Over 25 procedures per month

Q18 – On average, how many orthopaedic cases require radiology but do not get it (per week)?

Q19 – Do you have an inventory of your medical equipment?

1. No
2. Yes, kept manually
3. Yes, kept electronically

Q20 – Who is responsible for maintaining the equipment inventory?

Q21 – If your medical equipment breaks, what is the typical repair process?

Q22 – Full body lead vests in the operating room

1. Does your facility have full body lead vest(s) available in the operating room?
2. How many do you have?
3. What state are they in?

Q23 – Thyroid guards in the operating room

1. Does your facility have full body lead vest(s) available in the operating room?
2. How many do you have?
3. What state are they in?

Q24 – Eye protection in the operating room

1. Does your facility have full body lead vest(s) available in the operating room?
2. How many do you have?
3. What state are they in?

Q25 – Has your operating room staff been given a formal training on radiation safety?

Q26 – Has your operating room staff been given a formal training on ways to reduce radiation exposure?

Q27 – Has your operating room staff been given a formal training on C-arm machine use?

Q28 – Has your operating room staff received training on the importance of correct C-arm positioning to reduce radiation scatter?

Q29 – Is there biomedical technician training available at your facility?

Q30 – Has your operating room staff received training on the importance of correct C-arm positioning to reduce radiation scatter?

Q31 – How many Anaesthesiologists work at your facility?

1. Full time (5 days/week)
2. Part time (2-4 days/week)
3. Part time (1 day/week)
4. Haitian visiting surgeon
5. Foreign visiting surgeon

Q32 – How many Radiologists work at your facility?

1. Full time (5 days/week)
2. Part time (2-4 days/week)
3. Part time (1 day/week)
4. Haitian visiting surgeon
5. Foreign visiting surgeon

Q33 – How many Radiology technicians work at your facility?

1. Full time (5 days/week)
2. Part time (2-4 days/week)
3. Part time (1 day/week)
4. Haitian visiting surgeon
5. Foreign visiting surgeon

Q34 – If you employ radiology technicians at your facility, are they trained in intraoperative radiology positions and techniques?

Q35 – How many scrub nurses work at your facility?

1. Full time (5 days/week)
2. Part time (2-4 days/week)
3. Part time (1 day/week)
4. Haitian visiting surgeon
5. Foreign visiting surgeon

Q36 – How many operating rooms do you have at your facility?

Q37 – Are the operating rooms at your facility used regularly?

Q38 – How many operating rooms do you have at your facility?

Q39 – Do you have a C-arm machine at your facility?

Q40 – If you currently have a C-arm machine at our facility, is it functioning?

Q41 – How many C-arm imaging tables do you have at your facility?

Q42 – How many autoclaves do you have at your facility?

Q43 – How many X-ray machines do you have at your facility?

Q44 – How many CT scanners do you have at your facility?

Q45 – How many traction operating tables do you have at your facility?

Q46 – Does your facility have the ability to conduct routine maintenance and calibration of a C-arm machine?

Q47 – Does your facility have the financial ability to operate and maintain a C-arm?

**Supplementary material 2: Summary of variables within the five categories**

2.1 Staff category:

Essential subcategory: Orthopaedic surgeons, General surgeons, biomedical technicians, anaesthesiologists, radiologists, radiology technicians and scrub nurses.

Desired subcategory: Orthopaedic residents, paediatric surgeons, obstetric and gynaecology surgeons, Ear, Nose and Throat surgeons and other surgeons.

2.2 Space category:

Essential subcategory: operating rooms at the facility and whether the operating rooms are used regularly.

Desired subcategory: N/A

2.3 Stuff category:

Essential subcategory: X-ray with film processing, X-ray with CR processing, X-ray with digital processing, orthopaedic operating room table, orthopaedic drill and accessories, sterilizer for orthopaedic instruments, orthopaedics kits and trauma hardware, lead vests, thyroid guards, eye protection, C-arm imaging tables, autoclaves, X-ray machines, traction operating tables, access to electricity from city/village source, electricity from generator, solar for electricity, good internet connection, fuel and fuel storage to keep generator running and repair system for generator and/or solar system.

Desired subcategory: number of lead vests available, number of thyroid guards available and CT scanners available at the facility.

2.4 Systems category:

Essential subcategory: Ability to conduct routine maintenance and calibration of C-arm, inventory of medical equipment, repair process for medical equipment, radiation safety for operating room staff, education on reducing radiation exposure for operating room staff, formal training on C-arm for operating room staff, training on C-arm positioning to reduce radiation for operating room staff, biomedical technician training and financial ability to operate and maintain a C-arm machine.

Desired subcategory: inventory method and responsible of maintaining the inventory.

2.5 Surgical capacity category:

Essential subcategory: how many orthopaedic surgeries performed at the facility per month, how many of these procedures require imaging, how many cases require radiology but don’t receive it.

Desired subcategory: N/A
